# Does diabetes mellitus affect guided bone regeneration outcomes in individuals undergoing dental implants? A systematic review and meta-analysis

**DOI:** 10.3389/fdmed.2024.1352763

**Published:** 2024-01-24

**Authors:** Leandro Machado Oliveira, Fabricio Batistin Zanatta, Raquel Pippi Antoniazzi, Patrícia Almeida Miguez

**Affiliations:** ^1^ Department of Stomatology, Postgraduate Program in Dentistry - Periodontics, Universidade Federal de Santa Maria (UFSM), Santa Maria, Rio Grande do Sul, Brazil; ^2^ Division of Comprehensive Oral Health – Periodontology, Adams School of Dentistry, University of North Carolina at Chapel Hill, Chapel Hill, NC, United States

**Keywords:** alveolar ridge augmentation, dental implants, diabetes mellitus, systematic review, meta-analysis

## Abstract

**Objectives:**

This systematic review aimed to verify if diabetes affects vertical and horizontal ridge augmentation in individuals undergoing dental implant treatment with guided bone regeneration (GBR).

**Methods:**

Five databases were systematically screened up to September 2023, according to predefined eligibility criteria. The methodological risk of bias of the included studies was assessed using the ROBINS-I tool, and GRADE was used to evaluate the certainty of evidence. Random-effects pairwise meta-analyses were used to compare changes in vertical (height) and horizontal (width) alveolar bone dimensions between individuals exposed and unexposed to diabetes, through standardized mean differences (SMDs).

**Results:**

Three non-randomized controlled trials met the inclusion criteria, all of which showed a serious risk of bias. The results showed, with overall very low certainty on evidence, that individuals with diabetes did not exhibit a significant difference in horizontal (SMD = −0.41, 95% CI: −0.92–0.10) and vertical (SMD = 0.06, 95% CI: −0.43–0.56) ridge augmentation compared to the those without diabetes.

**Conclusions:**

The available evidence, albeit of very low certainty, suggests that diabetic individuals with moderate or good glycemic control undergoing dental implants and GBR show comparable horizontal and vertical bone gains to their unexposed counterparts.

**Systematic Review Registration:**

https://osf.io/bpx3t.

## Introduction

Diabetes mellitus (DM) is one of the foremost causes of mortality and disability in the world. The available evidence suggests that its prevalence is rising globally, primarily due to the increasing rates of obesity, driven by multiple contributing factors. Estimates from 2021 suggest a staggering 529 million individuals have diabetes, and this number is projected to double to around 1.31 billion in 2050 (1). On the spectrum of oral conditions and diseases, diabetes is a risk factor for periodontitis (2, 3), a condition pivotal in tooth loss (4). Individuals with diabetes are expected to experience higher rates of tooth loss and edentulism, regardless of their glycemic control status (5).

In this context, dental implants have emerged as a reliable option for the rehabilitation of partially or fully edentulous patients. Cumulative survival rates range from 90% to 96% after 10 years (6). Limited evidence supports no significant differences between diabetic and non-diabetic individuals. Notably, evidence in this regard is restricted to patients with well-controlled diabetes (7). Nevertheless, the increased susceptibility of diabetic patients to periodontitis and infection poses challenges for implant treatment due to pathological bone loss and tooth loss, resulting in both horizontal and vertical volume changes in the alveolar process pre- and post-tooth extraction. Adequate bone volume at the ideal prosthetic position is critical to achieving the desired function and aesthetics of implant-supported restorations (8).

To enhance dental implant therapy by reconstructing deficient alveolar ridges, various bone regenerative techniques have been assessed (9–13). Guided bone regeneration (GBR) is one such technique, which involves a membrane application to prevent non-osteogenic tissue interference in bone regeneration, closely mimicking the natural osteogenesis process (14). GBR can be implemented either before (staged technique) or simultaneously with implant placement (one-stage technique).

Despite advancements in biomaterials and surgery-related factors, knowledge gaps and uncertainties persist, particularly concerning patient-related factors influencing GBR outcomes (8). To address this, a comprehensive synthesis becomes imperative to investigate the current evidence regarding the impact of diabetes on GBR outcomes before or during implant placement. Hence, we conducted a systematic review to consolidate the existing evidence on this topic and evaluate its certainty level.

### Focused question

Is diabetes mellitus associated with reduced vertical and horizontal ridge augmentation in individuals receiving dental implant treatment and GBR?

## Materials and methods

This review is reported in accordance with the Preferred Reporting Items for Systematic Reviews and Meta-Analyses Statement checklist (PRISMA) (15). Methods were pre-specified in a protocol available elsewhere (https://osf.io/bpx3t) and were based on the Cochrane Handbook for Systematic Reviews of Interventions (16).

### Eligibility criteria

*Population:* Individuals aged 18 years or older requiring the placement of one or more implants in sites with horizontal and/or vertical ridge deficiencies necessitating GBR (one-stage or staged approaches) in either jaw. No restriction was imposed regarding techniques or employed biomaterials.

*Exposure:* Type 1 or type 2 diabetic individuals (based on study criteria);

*Comparator:* Individuals without a previous diabetes history;

*Outcomes:* The primary outcomes focused on changes in vertical (height) and/or horizontal (width) dimension of the ridge, assessed clinically or through cone-beam computed tomography (CBCT) using linear or volumetric measurements.

*Time:* At least four months of follow-up post-augmentation/implantation procedures;

*Study:* Either non-randomized or randomized controlled trials (N-RCT and RCT).

### Information sources and search strategy

We conducted a comprehensive and systematic search for relevant studies published up to 20 September 2023. The US National Library of Medicine (MEDLINE-PubMed), EMBASE, Cochrane Central Register of Controlled Trials (CENTRAL), and SCOPUS databases were investigated. We also searched Google Scholar (the first 300 most relevant hits) for grey literature. Additionally, the reference lists of the included studies were hand-searched to identify additional relevant studies. No language and date restrictions were imposed.

To construct our search strategy, we first consulted some systematic reviews addressing similar research questions to retrieve important search terms (11–13). Then, we applied a structured search strategy using a combination of controlled vocabulary (MeSH) and free text terms to retrieve relevant papers. The search strategies for the different databases are displayed in Appendix S1.

### Selection process

We performed a literature search using a two-step process by two independent reviewers (LMO and FBZ). Firstly, we screened record titles and abstracts retrieved from the searches. The Mendeley Desktop 1.19.4 (England) was used to manage the references and identify duplicates. Then, the retrieved records were classified as “include”, “exclude” or “uncertain”. Following this initial screening, we thoroughly read the full texts of papers identified as relevant. Studies satisfying all eligibility criteria were included and underwent data extraction. Divergences between the reviewers were resolved by discussion. In case of persistent disagreement, the judgment of an additional reviewer (RPA) was considered decisive.

### Data extraction and items

The same reviewers (LMO and FBZ) independently collected data from eligible studies using a pilot-tested Excel form (Microsoft, Redmond, Washington, United States). The collected data included study identification (first author, year of publication, and location); study design; sources of funding; duration; participants' characteristics (sex, age, smoking, and periodontal status); diabetes information (diagnosis); augmentation site and assessment method; surgical protocol; and baseline and follow-up measurements of vertical and horizontal ridge dimension.

### Risk-of-bias assessment

The risk of bias was assessed independently by the same two reviewers (LMO and FBZ) using the Risk of Bias in Non-Randomized Studies of Interventions (ROBINS-I) tool (20). Each study was graded according to seven domains: (1) confounding (especially sex, age, smoking, anatomical characteristics of the site of augmentation and [glycated hemoglobin (HbA1c) levels during the follow-up]; (2) selection of participants; (3) classification of interventions (considering potential misclassification due to the method of measuring diabetes); (4) deviations from intended interventions (exposures); (5) handling of missing data; (6) measurement of outcomes; and (7) selection of the reported result. Within each domain, we judged the risk of bias as “low” (comparable to a well-performed randomized clinical trial), “moderate” (sound for a non-randomized study), “serious” (indicating important concerns), “critical” (the study is too problematic to provide useful evidence) or “no information”. We thus rated the overall risk of bias for each study based on the most serious risk-of-bias judgment across any of the seven domains (overall risk of bias is serious if at least one domain is rated serious). Any disagreements were resolved through discussion. An additional reviewer's (RPA) judgment was considered decisive if a disagreement persisted. No automation tool was used. We created the summary risk-of-bias plot using the Risk-of-Bias VISualization tool (robvis).

### Data analysis

First, we summarized the data in evidence tables to outline the study characteristics. Thereafter, we conducted pair-wise comparisons using data from included studies to compare vertical and horizontal bone gain in diabetic and non-diabetic individuals. When change scores were not reported, we calculated by subtracting the final from baseline scores. We estimated the standard deviation (SD) changes using the formula SD_delta_ = √[(SD_baseline_)^2 ^+ (SD_final_)^2^ – (2*r*SD_baseline_*SD_final_)], in which we assumed *r* = 0.5 (16).

The included trials varied in protocols of dental implant placement and bone gain assessments. Thus, we used a random-effects model with the DerSimonian and Laird variance estimator (21) to determine the Hedges'g as standardized mean difference (SMD) and 95% confidence intervals (CI) as the summary estimate. All tests were two-tailed, with a significance level set at 0.05, except the Cochran's *Q* test (significance level at 0.1). We used the *I*^2^ statistic to assess heterogeneity, where values higher than 75% indicated high heterogeneity (16).

For outcomes with three or more studies, we estimated prediction intervals for pooled SMD to provide a range of expected effects. Additionally, sensitivity analyses were performed to identify any individual study significantly affecting the pooled result using the “leave one out” approach. All analyses were conducted using the Stata software, version 14.0 (Stata Corporation; College Station, TX, USA). However, due to the low number of included studies (<10), we were unable to explore heterogeneity with meta-regression and to quantify publication bias through statistical evaluation (Egger statistics).

### Certainty of the body of evidence

We assessed the certainty using the Grading of Recommendations Assessment, Development and Evaluation (GRADE) approach (22). We created a summary-of-findings table including the evidence profile (number of studies, assessed domains, and interpretation). Estimates of vertical and horizontal bone gain according to each comparison group were reported as SMD with 95% CI, along with the overall GRADE score.

The assessment of GRADE domains involved the levels of concern “serious”, “very serious” or “no concerns” for each domain. The key domains considered were risk of bias (according to ROBINS-I), inconsistency (evaluating heterogeneity in observed effects across studies and its potential explanations), imprecision (assessing whether confidence intervals led to conflicting interpretations), indirectness (differences between the characteristics of included studies and the research focused question), publication bias, and upgrading domains (according to the research focused question, only “large effect size” was considered).

## Results

**Figure 1:**
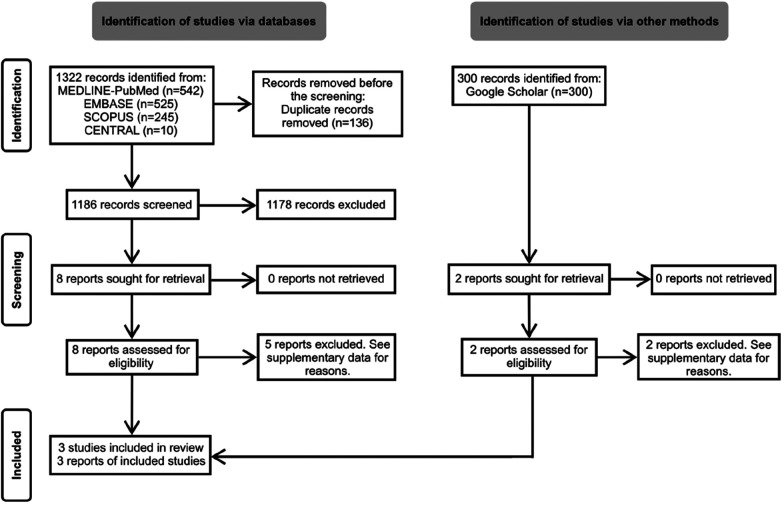
Flowchart of the selection process.

The search yielded 1,322 unique records. After screening titles and abstracts, we conducted detailed full-text readings of 10 preselected reports. Out of these, three non-randomized controlled trials (N-RCTs) met the eligibility criteria and were thus included in the review (17–19). The flow of references through the review is depicted in Figure 1. Additional details regarding excluded studies and the reasons for their exclusion are summarized in Appendix S2.

### Study characteristics

The included studies provided data of 42 individuals with type 2 diabetes and 45 individuals without. Current smokers were excluded in all three included trials. Specifically, two studies (18, 19) provided information on HbA1c levels of unexposed groups, while one study (17) provided details on diabetes duration. Diabetics were well controlled in two studies (18, 19) and moderately or well controlled in one (17). Additionally, one study clearly outlined the augmentation site (17). All patients across the studies received prophylactic antibiotic coverage and underwent similar regenerative procedures involving a 50% autologous bone and 50% synthetic bone substitute, associated with resorbable membranes. Notably, staged GBR was performed in two of the studies (17, 18).

The summary of findings using the evidence profile format is presented in Table 1.

**Table 1:** Main characteristics of included studies.

Identification Country Study design Funding Duration	Comparison groups (exposed, diabetic; control, non-diabetic)	Diabetes information	Site of augmentation Outcome measurements	Intervention
Erdogan et al., (17); Turkey; N-RCT; ITI Foundation; 5 months	Exposed: 12 (7♀/5♂) individuals with mean age (SD) of 52.6 (7.3); Control: 12 (5♀/7♂) individuals with mean age (SD) of 49.5 (9.3); Current smokers were excluded	Diagnosis of type 2 diabetes made at least five years earlier and confirmed by a physician; Patients on active treatment; Preoperative HbA1c levels between 6% and 7.5% (measured 1 week before the augmentation surgery); Duration of diabetes: 8.2 years (3.5); Initial mean HbA1c: 6.7% (0.3); HbA1c levels collected only from exposed individuals	Localized edentulous maxillary anterior or premolar region with at least 10 mm height (adequate) and <5 mm width (inadequate); CBCT (the buccal-palatal width at the 4 mm coronal aspect of the osseous crest on a line crossing the bisecting angle between the palatal and buccal cortical bones)	Prophylactic antibiotic coverage; Staged GBR; 50% autologous bone (mandibular ramus with bone scrapers) +50% synthetic bone substitute + resorbable membrane
De Angelis et al., (18); Italy; N-RCT; None; 6 months	Exposed: 12 (5♀/7♂) individuals with mean age (SD) of 66.0 (9.0); Control: 14 (7♀/7♂) individuals with mean age (SD) of 71.0 (7.0); Current smokers were excluded; Plaque and bleeding scores <15%	Diagnosis of type 2 diabetes confirmed by a physician; Exposed group HbA1c levels: <7%; Control group HbA1c levels: <5.7%	Upper or lower jaw; CBCT (details not reported)	Prophylactic antibiotic coverage; Staged GBR; 50% autologous bone (mandibular ramus with bone scrapers) + 50% synthetic bone substitute + resorbable membrane
De Angelis et al., (19); Italy; N-RCT; None; 6 months	Exposed: 18 (?♀/?♂) individuals with mean age (SD) of 68.22 (6.75); Control: 19 (?♀/?♂) individuals with mean age (SD) of 65.84 (8.64); Current smokers were excluded; Plaque and bleeding scores <15%	Diagnosis of type 2 diabetes confirmed by a physician; Exposed group HbA1c levels: <7%; Control group HbA1c levels: <5.7%	Upper or lower jaw; CBCT (details not reported)	Prophylactic antibiotic coverage; One-stage GBR; 50% autologous bone (mandibular ramus with bone scrapers) + 50% synthetic bone substitute + resorbable membrane

CBCT, cone beam computed tomography; GBR, guided bone regeneration; HbA1c, glycated hemoglobin; N-RCT, non-randomized controlled trials; SD, standard deviation.

### Pooled analyses

**Figure 2:**
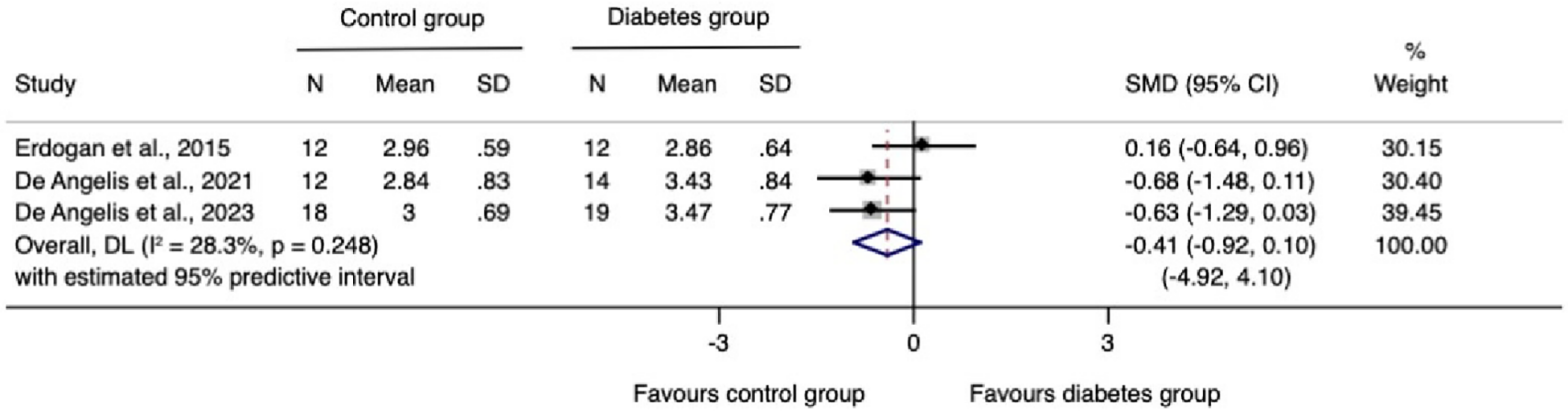
Meta-analysis for changes in the horizontal ridge dimension.

**Figure 3:**
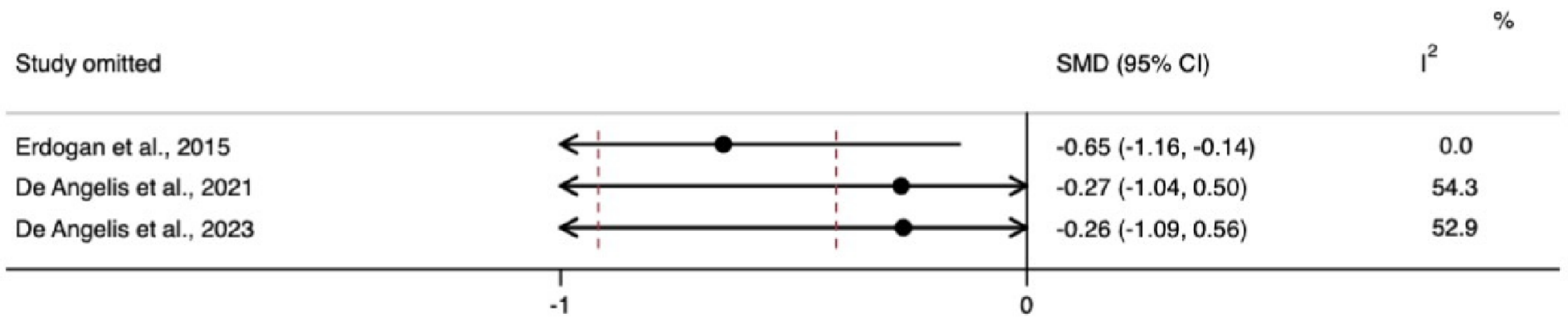
Sensitivity analysis (“leave-one-out”) for changes in the horizontal ridge dimension.

**Figure 4:**
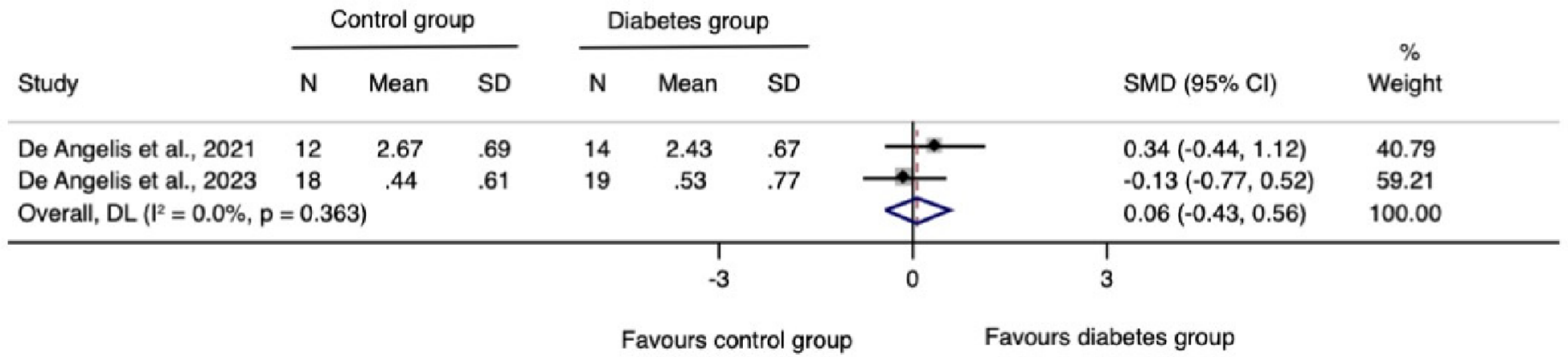
Meta-analysis for changes in the vertical ridge dimension.

The meta-analysis for changes in the horizontal ridge dimension contributed to data from all three N-RCTs (17–19). The pooled standardized mean difference calculated using a random-effects model showed no statistically significant difference between individuals with and without diabetes (SMD = −0.41, 95% CI: −0.92–0.10; *I*^2^ = 28.3%). Our prediction intervals suggested that in approximately 95% of all populations, the true effect size will fall within the range from −4.92 to 4.10, as illustrated in Figure 2. Sensitivity analysis revealed a key study (17), which was particularly influential in overall results. Removing this study from the analysis could lead to a statistically significant change in results, potentially favoring the unexposed group (Figure 3). Regarding the meta-analysis for changes in the vertical ridge dimension, the combined results from two studies (18, 19) did not show a statistically significant difference between exposed and unexposed individuals (SMD = 0.06, 95% CI: −0.43 to 0.56; *I*^2^ = 0.0%), as illustrated in Figure 4.

### Risk-of-bias assessment

**Figure 5:**
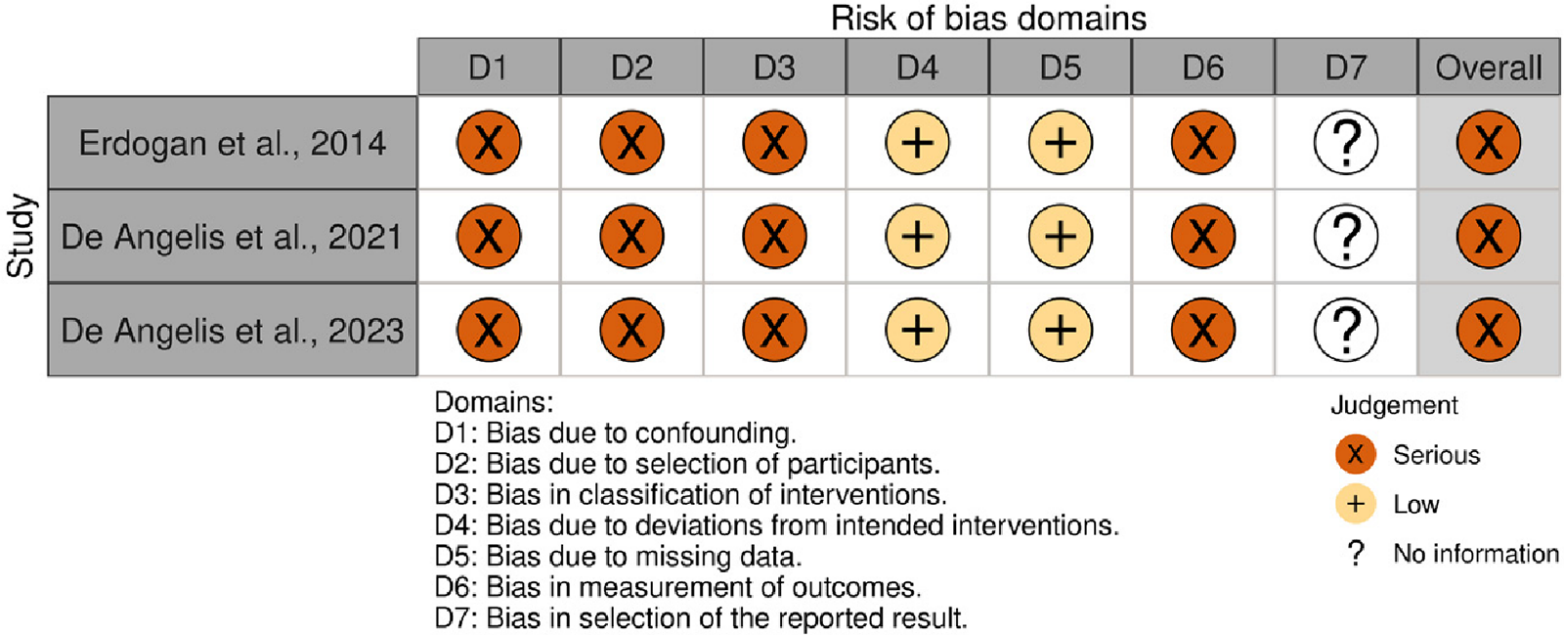
Risk-of-bias assessment.

All three studies exhibited a serious risk of bias. The lack of information regarding the HbA1c levels in the unexposed group (17), specific details about the site and anatomical characteristics of the site of augmentation (18, 19), and HbA1c levels during the follow-up period indicated unmeasured confounding (17–19). Additionally, using a single assessment to categorize HbA1c levels posed a risk of misclassifying hyperglycemia, as possible variations over time were not accounted for. The possibility of contamination of the unexposed group with non-diabetic hyperglycemic subjects cannot be ruled out. Lastly, the absence of blinding among outcome assessors led to our assessment of serious bias in outcome measurement (17–19). A summary of the results is presented in Figure 5.

### Summary of findings and assessment of certainty of the evidence

The summary of findings using the evidence profile format is presented in Table 2.

**Table 2:** Summary of findings on the influence of diabetes on guided bone regeneration outcomes in individuals undergoing dental implants.

Number of studies	Number of participants	Effect size	Risk of bias	Inconsistency	Indirectness	Imprecision	Impact	Certainty
Changes in the horizontal ridge dimension (standardized mean difference)								
3	87	−0.41 (−0.92–0.10)	Very serious^a^	Serious^b^	Not serious	Not serious	Individuals with diabetes undergoing dental implants and GBR show comparable horizontal bone gains to their counterparts	⊕○○○ Very low
Changes in the vertical ridge dimension (standardized mean difference)								
2	63	0.06 (−0.43–0.56)	Very serious^a^	Serious^b^	Not serious	Not serious	Individuals with diabetes undergoing dental implants and GBR show comparable vertical bone gains to their counterparts	⊕○○○ Very low

^a^ Downgraded due to a very serious risk of bias.

^b^ Downgraded due to serious inconsistency. Although there was no evidence of high heterogeneity in meta-analysis coefficients, it was not possible to explore whether between study differences affect the estimates.

## Discussion

This systematic review encompassed three N-RCTs (17–19) investigating peri-implant defect width and height changes following GBR performed either simultaneously or prior to implant placement, in individuals with and without type 2 diabetes mellitus. All three studies were considered for quantitative synthesis of changes in horizontal ridge dimensions, while two studies (18, 19) were included in the pooled estimates for vertical ridge augmentation. Our findings highlight a limited number of studies addressing this specific research question. Collectively, albeit of very low certainty evidence, the available evidence suggests that diabetic individuals with moderate or good glycemic control undergoing dental implants and GBR show comparable horizontal and vertical bone gains to their counterparts without diabetes.

Diabetes mellitus is associated with a spectrum of complications affecting the skeletal system and impairing bone regeneration, collectively known as “diabetic bone disease” or “diabetic osteopathy”. This condition is linked to the hyperglycemic environment during wound healing and the accumulation of advanced glycation end-products (AGEs) among other dysregulated cell functions (23). In the sensitivity analysis, we observed that bone width measurements in individuals with diabetes were significantly lower when a specific study was omitted. This omitted study did not provide blood glycemia level data for its control group. The absence of this information could have skewed the meta-analysis results, as it left the characteristics of the non-exposed group undefined. Conversely, when the meta-analysis included studies that clearly defined non-exposed individuals as those without diabetes, the results consistently showed that diabetics had worse bone-width outcomes, suggesting that diabetes may have a modifying effect on bone growth and repair. However, this result needs further confirmation from future primary studies. Pre-clinical studies have shown that GBR procedures can facilitate the regeneration of critical-size defects and promote new bone formation, even in the presence of uncontrolled diabetes. However, this process has been believed to be more predictable in individuals without diabetes or those with controlled diabetes (24, 25).

Additionally, diabetes may impair peri-implant bone formation and mineralization, resulting in compromised regeneration of peri-implant dehiscence defects (26, 27). Further studies have indicated that uncontrolled diabetes leads to a delayed and prolonged inflammatory response, accompanied by a downregulation of key genes and pathways essential for osteogenesis (24, 28). Some strategies have been proposed to mitigate the negative effects of uncontrolled diabetes during regenerative procedures, such as the use of hydrophilic micro-rough titanium surfaces, which may attenuate the pro-inflammatory response and restore macrophage homeostasis (29).

It is important to consider that diabetic patients undergoing GBR procedures may be moderate to severe periodontitis patients, and thus, likely patients with poor glycemic control leading to uncontrolled inflammation. These patients are expected to be going through regular periodontal recalls that can affect the level of inflammatory mediators as well as a possible remission of glucolipid metabolism and insulin resistance upon intervention (30). Previous SRs emphasize that significant changes in periodontal and HbA1c outcomes are detected after 3 months of periodontal treatment (31, 32). Thus, the importance of multiple HbA1c throughout periodontal and GBR procedures cannot be overstated in the journey to better understand the response to regenerative treatment. Further, diabetic patients often receive antibiotic therapy to help control microbiota and, thus, can influence glycemic control and inflammation which are relevant to wound healing (31). Exploration of the effects of antibiotics during the period of GBR healing has not been carried out previously.

It is important to highlight some limitations of this systematic review. We were unable to explore heterogeneity in meta-analyses using subgroups or meta-regression and investigate the presence of publication bias, since the number of included reports is restricted and reporting quality is limited. Additionally, our specific horizontal defect result seems not to be robust as indicated by the sensitivity analysis. It's important to emphasize that the available evidence is exclusively applicable to individuals with controlled type 2 diabetes. Extrapolating these findings to individuals with uncontrolled or type 1 diabetes is not warranted. Additionally, in light of the limited number of included studies and their low certainty of the evidence, additional evidence is imperative before formulating precise clinical recommendations.

### Implication for future studies

Considering the limited evidence addressing our focused question, it is imperative to conduct further investigations evaluating the impact of diabetes on GBR outcomes. Thus, future studies should improve on both reporting and methodological aspects. Considering that the defect anatomy predicts GBR outcomes (8), providing a comprehensive description of the augmentation site and detailed measurements is crucial. Additionally, continuous monitoring of HbA1c levels throughout the follow-up period is essential to evaluate the impact of metabolic control on outcomes. By embracing these best-practice methodologies and potentially integrating others, prospective research endeavors hold much promise when it comes to elevating the level of certainty in evidence, thereby contributing significantly to the delineation of clinical recommendations.

## Conclusion

Recognizing the limitations of this systematic review, the currently available evidence, albeit with very low certainty evidence, suggests there are no significant differences in peri-implant defect width and height changes following GBR, whether performed simultaneously or before implant placement, in subjects with and without controlled diabetes mellitus.

## Data Availability

The original contributions presented in the study are included in the article/Supplementary Material, further inquiries can be directed to the corresponding author.
