# Supplementary material for: Does diabetes mellitus affect guided bone regeneration outcomes in individuals undergoing dental implants? A systematic review and meta-analysis
Source: Front Dent Med. 2024 Jan 24;5:1352763. doi: 10.3389/fdmed.2024.1352763 (PMC11797803; doi:10.3389/fdmed.2024.1352763)
Supplement: Supplementary file 1 [file Table1.docx]

Appendix S1. Search strategies according to each database

| MEDLINE - PubMed |
| --- |
| ((((((((((((((((((((Diabetes Mellitus, Type 1[MeSH Terms]) OR (Autoimmune Diabetes)) OR (Diabetes Mellitus, Brittle)) OR (Diabetes Mellitus, Insulin-Dependent)) OR (Diabetes Mellitus, Insulin-Dependent, 1)) OR (Diabetes Mellitus, Juvenile-Onset)) OR (Diabetes Mellitus, Ketosis-Prone)) OR (Diabetes Mellitus, Sudden-Onset)) OR (Diabetes Mellitus, Type I)) OR (Diabetes, Autoimmune)) OR (IDDM)) OR (Insulin-Dependent Diabetes Mellitus 1)) OR (Juvenile-Onset Diabetes)) OR (Type 1 Diabetes)) OR (Type 1 Diabetes Mellitus)) OR ((((((((((((((((((Diabetes Mellitus, Type 2[MeSH Terms]) OR (Diabetes Mellitus, Adult-Onset)) OR (Diabetes Mellitus, Ketosis-Resistant)) OR (Diabetes Mellitus, Maturity-Onset)) OR (Diabetes Mellitus, Non Insulin Dependent)) OR (Diabetes Mellitus, Non-Insulin-Dependent)) OR (Diabetes Mellitus, Noninsulin Dependent)) OR (Diabetes Mellitus, Noninsulin-Dependent)) OR (Diabetes Mellitus, Slow-Onset)) OR (Diabetes Mellitus, Stable)) OR (Diabetes Mellitus, Type II)) OR (MODY)) OR (Maturity-Onset Diabetes)) OR (Maturity-Onset Diabetes Mellitus)) OR (NIDDM)) OR (Noninsulin-Dependent Diabetes Mellitus)) OR (Type 2 Diabetes)) OR (Type 2 Diabetes Mellitus))) OR (Diabetes Mellitus[MeSH Terms])) OR (hb1ac)) OR (Hyperglycemia[MeSH Terms])) OR (Diabetes)) AND (((((Bone Regeneration[MeSH Terms]) OR (Alveolar Bone Grafting[MeSH Terms])) OR (((Alveolar Ridge Augmentation[MeSH Terms]) OR (Mandibular Ridge Augmentation)) OR (Maxillary Ridge Augmentation))) OR (((((((Bone Substitutes[MeSH Terms]) OR (Bone Replacement Material)) OR (Bone Replacement Materials)) OR (Bone Substitute)) OR (Replacement Material, Bone)) OR (Replacement Materials, Bone)) OR (Substitutes, Bone))) OR (Guided bone regeneration)) |
| EMBASE |
| ('bone regeneration'/exp OR 'bone regeneration' OR 'regeneration, bone' OR 'alveolar bone grafting'/exp OR 'alveolar bone grafting' OR 'alveolar ridge augmentation'/exp OR 'alveolar ridge augmentation' OR 'ridge augmentation procedure' OR 'bone prosthesis'/exp OR 'hydroset' OR 'bone endoprosthesis' OR 'bone prosthesis' OR 'bone prosthesis (physical object)' OR 'bone substitute' OR 'bone substitutes' OR 'guided bone regeneration'/exp) AND ('tooth implant'/exp OR 'bicon' OR 'grafton' OR 'straumann mini' OR 'straumann pure' OR 'swish active' OR 'swish tapered' OR 'variobase' OR 'dental implant' OR 'dental implants' OR 'implant, teeth' OR 'implant, tooth' OR 'implants, teeth' OR 'implants, tooth' OR 'intramucosal dental implant' OR 'teeth implant' OR 'teeth implants' OR 'tooth implant' OR 'tooth implants' OR 'tooth implantation'/exp OR 'blade implantation' OR 'dental implantation' OR 'dental implantation, endosseous' OR 'dental implantation, endosseous, endodontic' OR 'dental implantation, subperiosteal' OR 'endodontic endosseous dental implantation' OR 'endosseous dental implantation' OR 'immediate dental implant loading' OR 'subperiosteal dental implantation' OR 'tooth implantation' OR 'implant'/exp OR 'biomedical implant' OR 'implant' OR 'implant material' OR 'implantable material' OR 'implantation material' OR 'implants' OR 'surgical implant') AND ('diabetes mellitus'/exp OR 'diabetes' OR 'diabetes mellitus' OR 'diabetic' OR 'non insulin dependent diabetes mellitus'/exp OR 'niddm (non insulin dependent diabetes mellitus)' OR 't2dm' OR 'adult onset diabetes' OR 'adult onset diabetes mellitus' OR 'diabetes mellitus type 2' OR 'diabetes mellitus type ii' OR 'diabetes mellitus, maturity onset' OR 'diabetes mellitus, non insulin dependent' OR 'diabetes mellitus, non-insulin-dependent' OR 'diabetes mellitus, type 2' OR 'diabetes mellitus, type ii' OR 'diabetes type 2' OR 'diabetes type ii' OR 'diabetes, adult onset' OR 'dm 2' OR 'insulin independent diabetes' OR 'insulin independent diabetes mellitus' OR 'ketosis resistant diabetes mellitus' OR 'maturity onset diabetes' OR 'maturity onset diabetes mellitus' OR 'maturity onset diabetes of the young' OR 'niddm' OR 'non insulin dependent diabetes' OR 'non insulin dependent diabetes mellitus' OR 'non-insulin-dependent diabetes mellitus' OR 'noninsulin dependent diabetes' OR 'noninsulin dependent diabetes mellitus' OR 'type 2 diabetes' OR 'type 2 diabetes mellitus' OR 'type ii diabetes' OR 'type ii diabetes mellitus' OR 'insulin dependent diabetes mellitus'/exp OR 't1dm' OR 'brittle diabetes' OR 'brittle diabetes mellitus' OR 'diabetes mellitus type 1' OR 'diabetes mellitus type i' OR 'diabetes mellitus, brittle' OR 'diabetes mellitus, insulin dependent' OR 'diabetes mellitus, insulin-dependent' OR 'diabetes mellitus, juvenile onset' OR 'diabetes mellitus, type 1' OR 'diabetes mellitus, type i' OR 'diabetes type 1' OR 'diabetes type i' OR 'diabetes, juvenile' OR 'dm 1' OR 'early onset diabetes mellitus' OR 'iddm' OR 'insulin dependent diabetes' OR 'insulin dependent diabetes mellitus' OR 'insulin-dependent diabetes mellitus' OR 'juvenile diabetes' OR 'juvenile diabetes mellitus' OR 'juvenile onset diabetes' OR 'juvenile onset diabetes mellitus' OR 'ketoacidotic diabetes' OR 'labile diabetes mellitus' OR 'mckusick 22210' OR 'type 1 diabetes' OR 'type 1 diabetes mellitus' OR 'type i diabetes' OR 'type i diabetes mellitus' OR 'hyperglycemia'/exp OR 'glucose blood level, elevated' OR 'glycemia, hyper' OR 'hyperglucemia' OR 'hyperglycaemia' OR 'hyperglycemia' OR 'hyperglycemic syndrome' OR 'glycated hemoglobin'/exp OR 'glycated haemoglobin' OR 'glycated hemoglobin' OR 'glycated hemoglobin a' OR 'glycohaemoglobin' OR 'glycohemoglobin' OR 'glycosyl haemoglobin' OR 'glycosyl hemoglobin' OR 'glycosylated haemoglobin' OR 'glycosylated hemoglobin' OR 'glycosylhaemoglobin' OR 'glycosylhemoglobin' OR 'glycosylised haemoglobin' OR 'glycosylized hemoglobin' OR 'haemoglobin a1' OR 'haemoglobin a 1' OR 'haemoglobin a, glycosylated' OR 'haemoglobin ai' OR 'haemoglobin alpha 1' OR 'haemoglobin glycoside' OR 'haemoglobin glycosylation' OR 'hemoglobin a, glycosylated' OR 'hemoglobin glycoside') |
| CENTRAL |
| #1 MeSH descriptor: [Diabetes Mellitus] explode all trees  #2 MeSH descriptor: [Diabetes Mellitus, Type 2] explode all trees  #3 MeSH descriptor: [Diabetes Mellitus, Type 1] explode all trees  #4 MeSH descriptor: [Bone Regeneration] explode all trees  #5 MeSH descriptor: [Alveolar Bone Grafting] explode all trees  #6 MeSH descriptor: [Alveolar Ridge Augmentation] explode all trees  #7 MeSH descriptor: [Bone Substitutes] explode all trees  #8 Guided bone regeneration  #9 #1 OR #2 OR #3  #10 #4 OR #5 OR #6 OR #7 OR #8  #11 #9 AND #10 |
| SCOPUS |
| ( TITLE-ABS-KEY ( diabetes ) ) AND ( ( TITLE-ABS-KEY ( bone AND regeneration ) ) OR ( TITLE-ABS-KEY ( alveolar AND bone AND grafting ) ) OR ( TITLE-ABS-KEY ( alveolar AND ridge AND augmentation ) ) OR ( TITLE-ABS-KEY ( bone AND substitutes ) ) ) AND ( TITLE-ABS-KEY ( implant ) ) |
| Google Scholar |
| (("diabetes") OR ("hyperglycemia") OR ("hyperglycaemia")) AND (("horizontal") OR ("width") OR ("vertical") OR ("height")) AND ("guided bone regeneration") AND ("implant") |

Appendix S2. Excluded reports and reasons

| Excluded reports | Reasons |
| --- | --- |
| (Ghanem et al., 2022; Zhang et al., 2019) | Studies evaluating extraction sockets |
| (Abdulwassie & Dhanrajani, 2002; Farzad et al., 2002; Tawil et al., 2008; Turkyilmaz, 2010) | Lack of control group |
| (Schwartz-Arad et al., 2005) | The regeneration procedure was onlay bone graph. |
